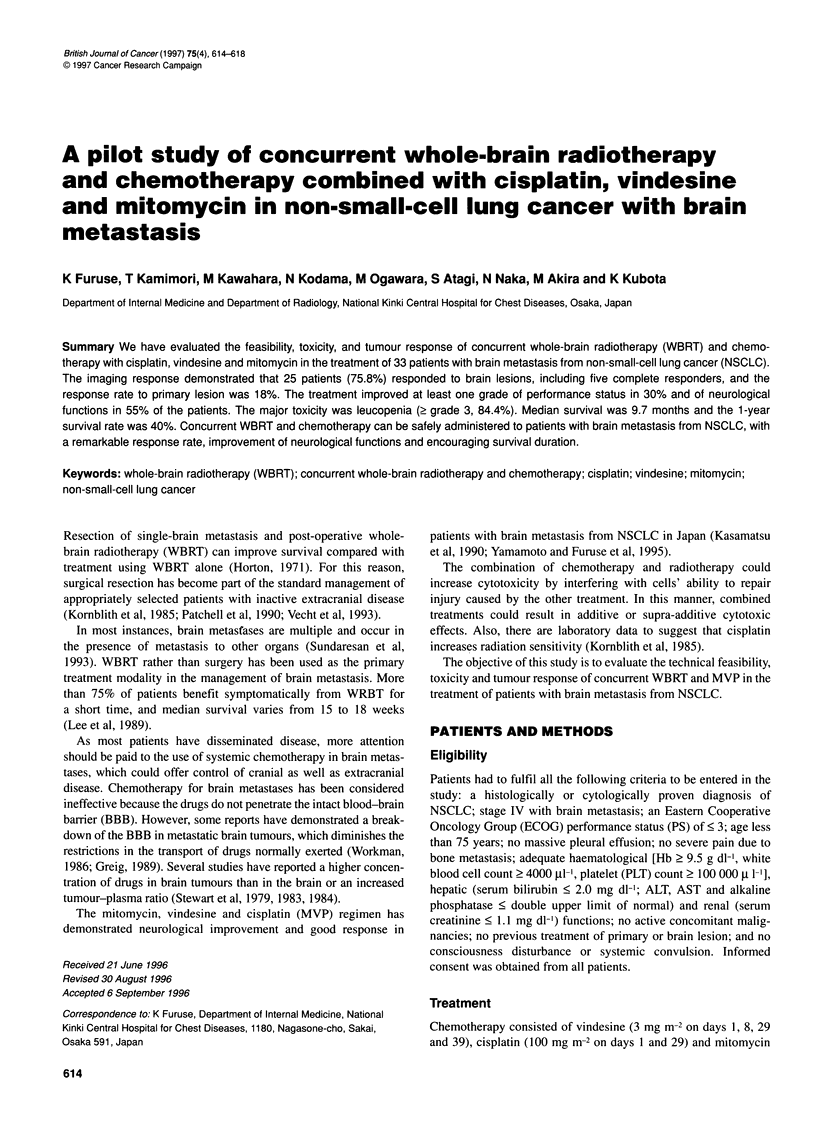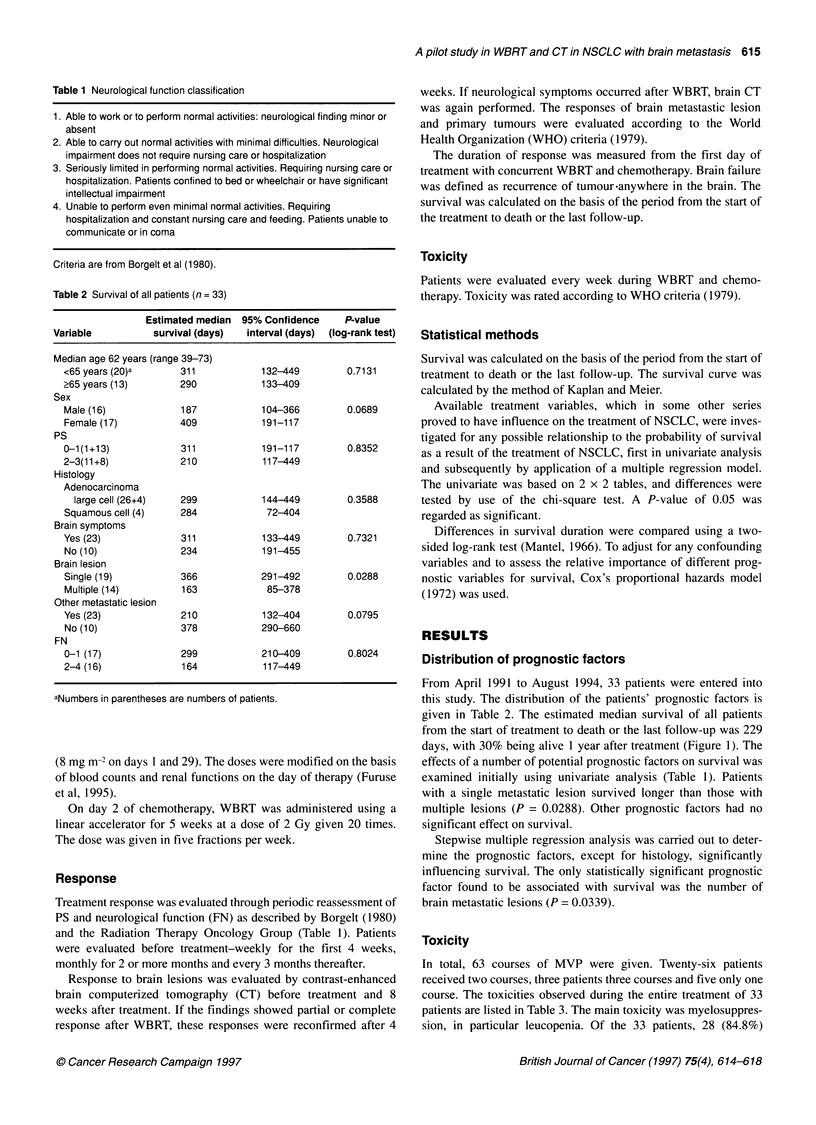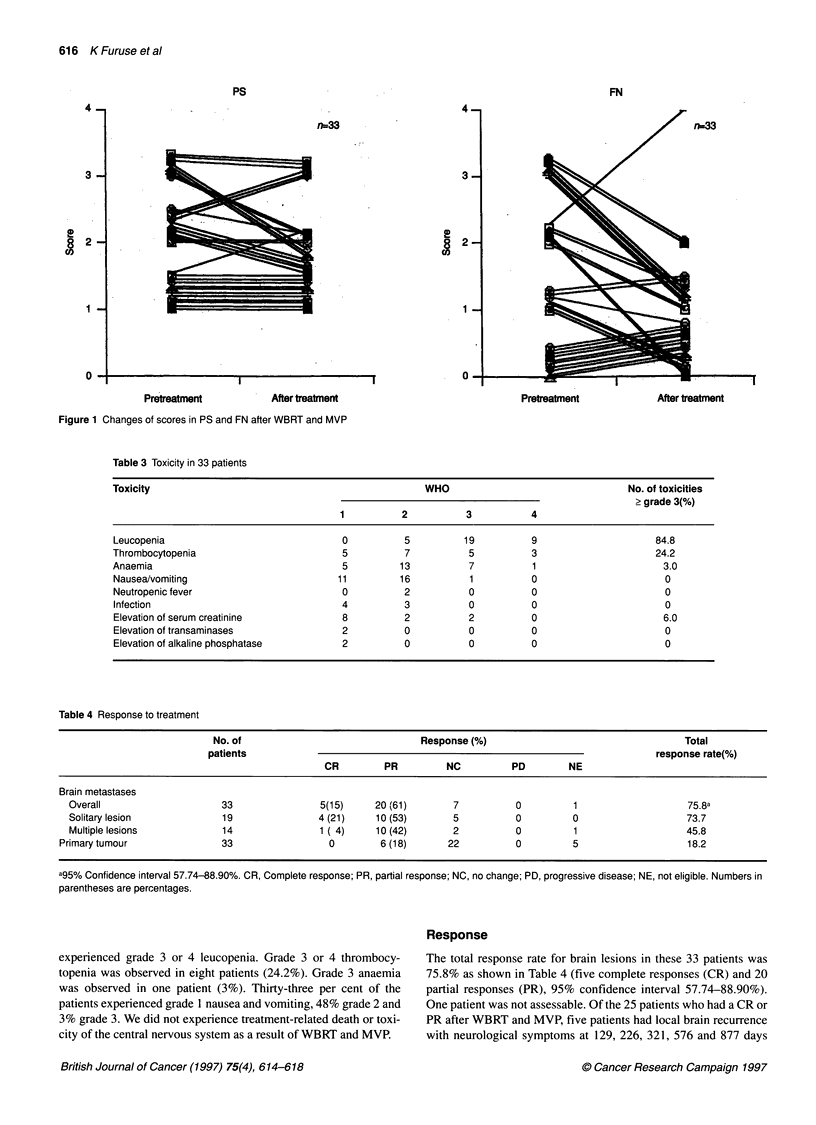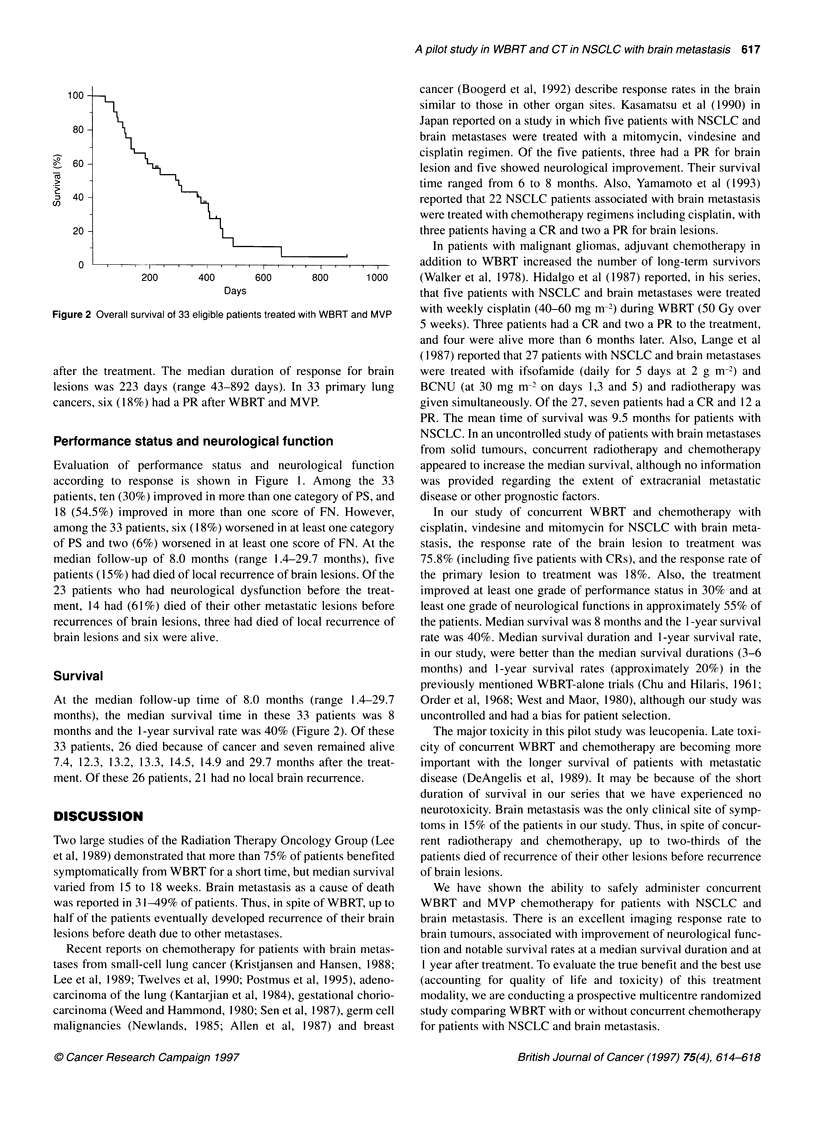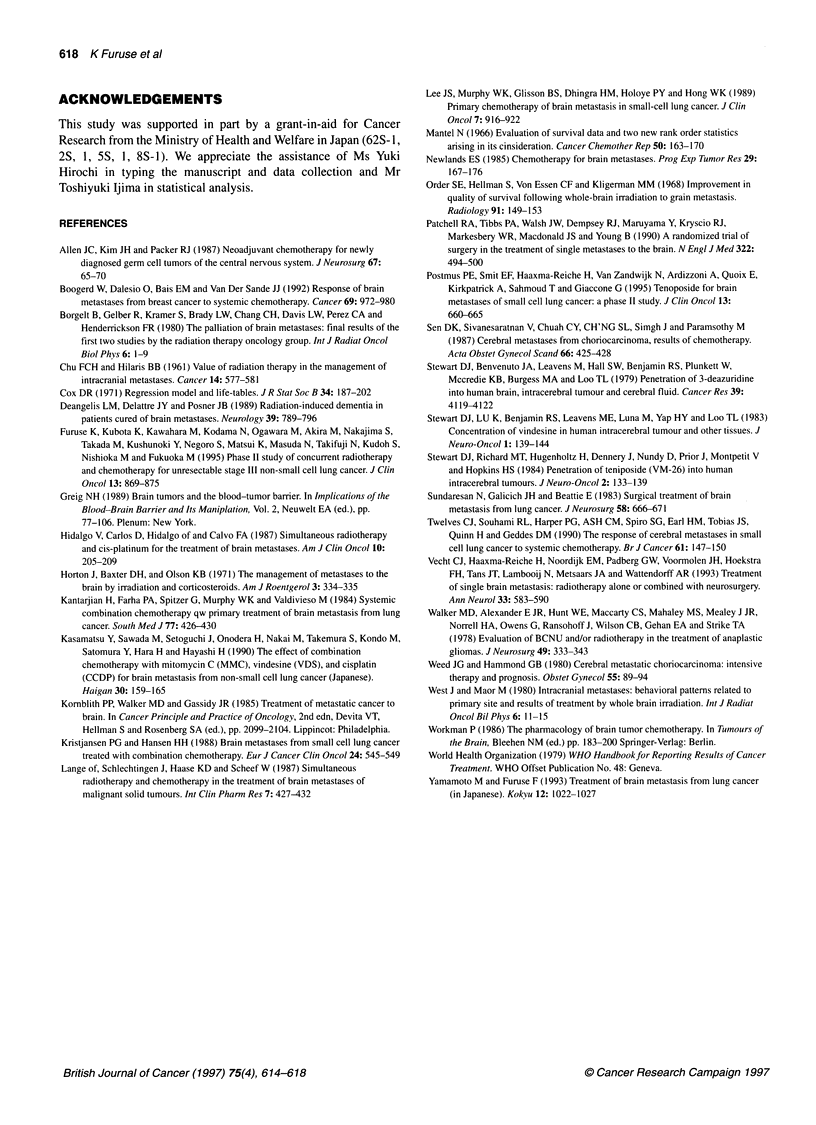# A pilot study of concurrent whole-brain radiotherapy and chemotherapy combined with cisplatin, vindesine and mitomycin in non-small-cell lung cancer with brain metastasis.

**DOI:** 10.1038/bjc.1997.108

**Published:** 1997

**Authors:** K. Furuse, T. Kamimori, M. Kawahara, N. Kodama, M. Ogawara, S. Atagi, N. Naka, M. Akira, K. Kubota

**Affiliations:** Department of Internal Medicine, National Kinki Central Hospital for Chest Diseases, Osaka, Japan.

## Abstract

We have evaluated the feasibility, toxicity, and tumour response of concurrent whole-brain radiotherapy (WBRT) and chemotherapy with cisplatin, vindesine and mitomycin in the treatment of 33 patients with brain metastasis from non-small-cell lung cancer (NSCLC). The imaging response demonstrated that 25 patients (75.8%) responded to brain lesions, including five complete responders, and the response rate to primary lesion was 18%. The treatment improved at least one grade of performance status in 30% and of neurological functions in 55% of the patients. The major toxicity was leucopenia (> or = grade 3, 84.4%). Median survival was 9.7 months and the 1-year survival rate was 40%. Concurrent WBRT and chemotherapy can be safely administered to patients with brain metastasis from NSCLC, with a remarkable response rate, improvement of neurological functions and encouraging survival duration.


British Journal of Cancer (1997) 75(4), 614–618
© 1997 Cancer Research Campaign

### A pilot study of concurrent whole-brain radiotherapy and chemotherapy combined with cisplatin, vindesine and mitomycin in non-small-cell lung cancer with brain metastasis

**K Furuse, T Kamimori, M Kawahara, N Kodama, M Ogawara, S Atagi, N Naka, M Akira and K Kubota**

Department of Internal Medicine and Department of Radiology, National Kinki Central Hospital for Chest Diseases, Osaka, Japan

**Summary** We have evaluated the feasibility, toxicity, and tumour response of concurrent whole-brain radiotherapy (WBRT) and chemotherapy with cisplatin, vindesine and mitomycin in the treatment of 33 patients with brain metastasis from non-small-cell lung cancer (NSCLC). The imaging response demonstrated that 25 patients (75.8%) responded to brain lesions, including five complete responders, and the response rate to primary lesion was 18%. The treatment improved at least one grade of performance status in 30% and of neurological functions in 55% of the patients. The major toxicity was leucopenia (≥ grade 3, 84.4%). Median survival was 9.7 months and the 1-year survival rate was 40%. Concurrent WBRT and chemotherapy can be safely administered to patients with brain metastasis from NSCLC, with a remarkable response rate, improvement of neurological functions and encouraging survival duration.

**Keywords:** whole-brain radiotherapy (WBRT); concurrent whole-brain radiotherapy and chemotherapy; cisplatin; vindesine; mitomycin; non-small-cell lung cancer

Resection of single-brain metastasis and post-operative whole-brain radiotherapy (WBRT) can improve survival compared with treatment using WBRT alone (Horton, 1971). For this reason, surgical resection has become part of the standard management of appropriately selected patients with inactive extracranial disease (Kornblith et al, 1985; Patchell et al, 1990; Vecht et al, 1993).

In most instances, brain metasfases are multiple and occur in the presence of metastasis to other organs (Sundaresan et al, 1993). WBRT rather than surgery has been used as the primary treatment modality in the management of brain metastasis. More than 75% of patients benefit symptomatically from WRBT for a short time, and median survival varies from 15 to 18 weeks (Lee et al, 1989).

As most patients have disseminated disease, more attention should be paid to the use of systemic chemotherapy in brain metastases, which could offer control of cranial as well as extracranial disease. Chemotherapy for brain metastases has been considered ineffective because the drugs do not penetrate the intact blood–brain barrier (BBB). However, some reports have demonstrated a breakdown of the BBB in metastatic brain tumours, which diminishes the restrictions in the transport of drugs normally exerted (Workman, 1986; Greig, 1989). Several studies have reported a higher concentration of drugs in brain tumours than in the brain or an increased tumour–plasma ratio (Stewart et al, 1979, 1983, 1984).

The mitomycin, vindesine and cisplatin (MVP) regimen has demonstrated neurological improvement and good response in patients with brain metastasis from NSCLC in Japan (Kasamatsu et al, 1990; Yamamoto and Furuse et al, 1995).

The combination of chemotherapy and radiotherapy could increase cytotoxicity by interfering with cells' ability to repair injury caused by the other treatment. In this manner, combined treatments could result in additive or supra-additive cytotoxic effects. Also, there are laboratory data to suggest that cisplatin increases radiation sensitivity (Kornblith et al, 1985).

The objective of this study is to evaluate the technical feasibility, toxicity and tumour response of concurrent WBRT and MVP in the treatment of patients with brain metastasis from NSCLC.

### PATIENTS AND METHODS

### Eligibility

Patients had to fulfil all the following criteria to be entered in the study: a histologically or cytologically proven diagnosis of NSCLC; stage IV with brain metastasis; an Eastern Cooperative Oncology Group (ECOG) performance status (PS) of ≤ 3; age less than 75 years; no massive pleural effusion; no severe pain due to bone metastasis; adequate haematological [Hb ≥ 9.5 g $dl^{-1}$, white blood cell count ≥ 4000 $\mu l^{-1}$, platelet (PLT) count ≥ 100 000 $\mu\ l^{-1}$], hepatic (serum bilirubin ≤ 2.0 mg $dl^{-1}$; ALT, AST and alkaline phosphatase ≤ double upper limit of normal) and renal (serum creatinine ≤ 1.1 mg $dl^{-1}$) functions; no active concomitant malignancies; no previous treatment of primary or brain lesion; and no consciousness disturbance or systemic convulsion. Informed consent was obtained from all patients.

### Treatment

Chemotherapy consisted of vindesine (3 mg $m^{-2}$ on days 1, 8, 29 and 39), cisplatin (100 mg $m^{-2}$ on days 1 and 29) and mitomycin

*Received 21 June 1996*
*Revised 30 August 1996*
*Accepted 6 September 1996*

*Correspondence to:* K Furuse, Department of Internal Medicine, National Kinki Central Hospital for Chest Diseases, 1180, Nagasone-cho, Sakai, Osaka 591, Japan

Table 1 Neurological function classification

1. Able to work or to perform normal activities: neurological finding minor or absent
2. Able to carry out normal activities with minimal difficulties. Neurological impairment does not require nursing care or hospitalization
3. Seriously limited in performing normal activities. Requiring nursing care or hospitalization. Patients confined to bed or wheelchair or have significant intellectual impairment
4. Unable to perform even minimal normal activities. Requiring hospitalization and constant nursing care and feeding. Patients unable to communicate or in coma

Criteria are from Borgelt et al (1980).

Table 2 Survival of all patients ($n$ = 33)

| Variable | Estimated median survival (days) | 95% Confidence interval (days) | *P*-value (log-rank test) |
|---|---|---|---|
| Median age 62 years (range 39–73) | | | |
| <65 years (20)[a] | 311 | 132–449 | 0.7131 |
| ≥65 years (13) | 290 | 133–409 | |
| Sex | | | |
| Male (16) | 187 | 104–366 | 0.0689 |
| Female (17) | 409 | 191–117 | |
| PS | | | |
| 0–1(1+13) | 311 | 191–117 | 0.8352 |
| 2–3(11+8) | 210 | 117–449 | |
| Histology | | | |
| Adenocarcinoma large cell (26+4) | 299 | 144–449 | 0.3588 |
| Squamous cell (4) | 284 | 72–404 | |
| Brain symptoms | | | |
| Yes (23) | 311 | 133–449 | 0.7321 |
| No (10) | 234 | 191–455 | |
| Brain lesion | | | |
| Single (19) | 366 | 291–492 | 0.0288 |
| Multiple (14) | 163 | 85–378 | |
| Other metastatic lesion | | | |
| Yes (23) | 210 | 132–404 | 0.0795 |
| No (10) | 378 | 290–660 | |
| FN | | | |
| 0–1 (17) | 299 | 210–409 | 0.8024 |
| 2–4 (16) | 164 | 117–449 | |

[a]Numbers in parentheses are numbers of patients.

(8 mg m$^{-2}$ on days 1 and 29). The doses were modified on the basis of blood counts and renal functions on the day of therapy (Furuse et al, 1995).

On day 2 of chemotherapy, WBRT was administered using a linear accelerator for 5 weeks at a dose of 2 Gy given 20 times. The dose was given in five fractions per week.

### Response

Treatment response was evaluated through periodic reassessment of PS and neurological function (FN) as described by Borgelt (1980) and the Radiation Therapy Oncology Group (Table 1). Patients were evaluated before treatment–weekly for the first 4 weeks, monthly for 2 or more months and every 3 months thereafter.

Response to brain lesions was evaluated by contrast-enhanced brain computerized tomography (CT) before treatment and 8 weeks after treatment. If the findings showed partial or complete response after WBRT, these responses were reconfirmed after 4 weeks. If neurological symptoms occurred after WBRT, brain CT was again performed. The responses of brain metastatic lesion and primary tumours were evaluated according to the World Health Organization (WHO) criteria (1979).

The duration of response was measured from the first day of treatment with concurrent WBRT and chemotherapy. Brain failure was defined as recurrence of tumour·anywhere in the brain. The survival was calculated on the basis of the period from the start of the treatment to death or the last follow-up.

### Toxicity

Patients were evaluated every week during WBRT and chemotherapy. Toxicity was rated according to WHO criteria (1979).

### Statistical methods

Survival was calculated on the basis of the period from the start of treatment to death or the last follow-up. The survival curve was calculated by the method of Kaplan and Meier.

Available treatment variables, which in some other series proved to have influence on the treatment of NSCLC, were investigated for any possible relationship to the probability of survival as a result of the treatment of NSCLC, first in univariate analysis and subsequently by application of a multiple regression model. The univariate was based on 2 × 2 tables, and differences were tested by use of the chi-square test. A *P*-value of 0.05 was regarded as significant.

Differences in survival duration were compared using a two-sided log-rank test (Mantel, 1966). To adjust for any confounding variables and to assess the relative importance of different prognostic variables for survival, Cox's proportional hazards model (1972) was used.

### RESULTS

### Distribution of prognostic factors

From April 1991 to August 1994, 33 patients were entered into this study. The distribution of the patients' prognostic factors is given in Table 2. The estimated median survival of all patients from the start of treatment to death or the last follow-up was 229 days, with 30% being alive 1 year after treatment (Figure 1). The effects of a number of potential prognostic factors on survival was examined initially using univariate analysis (Table 1). Patients with a single metastatic lesion survived longer than those with multiple lesions ($P$ = 0.0288). Other prognostic factors had no significant effect on survival.

Stepwise multiple regression analysis was carried out to determine the prognostic factors, except for histology, significantly influencing survival. The only statistically significant prognostic factor found to be associated with survival was the number of brain metastatic lesions ($P$ = 0.0339).

### Toxicity

In total, 63 courses of MVP were given. Twenty-six patients received two courses, three patients three courses and five only one course. The toxicities observed during the entire treatment of 33 patients are listed in Table 3. The main toxicity was myelosuppression, in particular leucopenia. Of the 33 patients, 28 (84.8%)

© Cancer Research Campaign 1997

**Figure 1** Changes of scores in PS and FN after WBRT and MVP

**Table 3** Toxicity in 33 patients

| Toxicity | WHO | | | | No. of toxicities ≥ grade 3(%) |
|---|---|---|---|---|---|
| | 1 | 2 | 3 | 4 | |
| Leucopenia | 0 | 5 | 19 | 9 | 84.8 |
| Thrombocytopenia | 5 | 7 | 5 | 3 | 24.2 |
| Anaemia | 5 | 13 | 7 | 1 | 3.0 |
| Nausea/vomiting | 11 | 16 | 1 | 0 | 0 |
| Neutropenic fever | 0 | 2 | 0 | 0 | 0 |
| Infection | 4 | 3 | 0 | 0 | 0 |
| Elevation of serum creatinine | 8 | 2 | 2 | 0 | 6.0 |
| Elevation of transaminases | 2 | 0 | 0 | 0 | 0 |
| Elevation of alkaline phosphatase | 2 | 0 | 0 | 0 | 0 |

**Table 4** Response to treatment

| | No. of patients | Response (%) | | | | | Total response rate(%) |
|---|---|---|---|---|---|---|---|
| | | CR | PR | NC | PD | NE | |
| Brain metastases | | | | | | | |
| Overall | 33 | 5(15) | 20 (61) | 7 | 0 | 1 | 75.8[a] |
| Solitary lesion | 19 | 4 (21) | 10 (53) | 5 | 0 | 0 | 73.7 |
| Multiple lesions | 14 | 1 ( 4) | 10 (42) | 2 | 0 | 1 | 45.8 |
| Primary tumour | 33 | 0 | 6 (18) | 22 | 0 | 5 | 18.2 |

[a]95% Confidence interval 57.74–88.90%. CR, Complete response; PR, partial response; NC, no change; PD, progressive disease; NE, not eligible. Numbers in parentheses are percentages.

experienced grade 3 or 4 leucopenia. Grade 3 or 4 thrombocytopenia was observed in eight patients (24.2%). Grade 3 anaemia was observed in one patient (3%). Thirty-three per cent of the patients experienced grade 1 nausea and vomiting, 48% grade 2 and 3% grade 3. We did not experience treatment-related death or toxicity of the central nervous system as a result of WBRT and MVP.

### Response

The total response rate for brain lesions in these 33 patients was 75.8% as shown in Table 4 (five complete responses (CR) and 20 partial responses (PR), 95% confidence interval 57.74–88.90%). One patient was not assessable. Of the 25 patients who had a CR or PR after WBRT and MVP, five patients had local brain recurrence with neurological symptoms at 129, 226, 321, 576 and 877 days

© Cancer Research Campaign 1997

**Figure 2** Overall survival of 33 eligible patients treated with WBRT and MVP

after the treatment. The median duration of response for brain lesions was 223 days (range 43–892 days). In 33 primary lung cancers, six (18%) had a PR after WBRT and MVP.

### Performance status and neurological function

Evaluation of performance status and neurological function according to response is shown in Figure 1. Among the 33 patients, ten (30%) improved in more than one category of PS, and 18 (54.5%) improved in more than one score of FN. However, among the 33 patients, six (18%) worsened in at least one category of PS and two (6%) worsened in at least one score of FN. At the median follow-up of 8.0 months (range 1.4–29.7 months), five patients (15%) had died of local recurrence of brain lesions. Of the 23 patients who had neurological dysfunction before the treatment, 14 had (61%) died of their other metastatic lesions before recurrences of brain lesions, three had died of local recurrence of brain lesions and six were alive.

### Survival

At the median follow-up time of 8.0 months (range 1.4–29.7 months), the median survival time in these 33 patients was 8 months and the 1-year survival rate was 40% (Figure 2). Of these 33 patients, 26 died because of cancer and seven remained alive 7.4, 12.3, 13.2, 13.3, 14.5, 14.9 and 29.7 months after the treatment. Of these 26 patients, 21 had no local brain recurrence.

### DISCUSSION

Two large studies of the Radiation Therapy Oncology Group (Lee et al, 1989) demonstrated that more than 75% of patients benefited symptomatically from WBRT for a short time, but median survival varied from 15 to 18 weeks. Brain metastasis as a cause of death was reported in 31–49% of patients. Thus, in spite of WBRT, up to half of the patients eventually developed recurrence of their brain lesions before death due to other metastases.

Recent reports on chemotherapy for patients with brain metastases from small-cell lung cancer (Kristjansen and Hansen, 1988; Lee et al, 1989; Twelves et al, 1990; Postmus et al, 1995), adenocarcinoma of the lung (Kantarjian et al, 1984), gestational choriocarcinoma (Weed and Hammond, 1980; Sen et al, 1987), germ cell malignancies (Newlands, 1985; Allen et al, 1987) and breast cancer (Boogerd et al, 1992) describe response rates in the brain similar to those in other organ sites. Kasamatsu et al (1990) in Japan reported on a study in which five patients with NSCLC and brain metastases were treated with a mitomycin, vindesine and cisplatin regimen. Of the five patients, three had a PR for brain lesion and five showed neurological improvement. Their survival time ranged from 6 to 8 months. Also, Yamamoto et al (1993) reported that 22 NSCLC patients associated with brain metastasis were treated with chemotherapy regimens including cisplatin, with three patients having a CR and two a PR for brain lesions.

In patients with malignant gliomas, adjuvant chemotherapy in addition to WBRT increased the number of long-term survivors (Walker et al, 1978). Hidalgo et al (1987) reported, in his series, that five patients with NSCLC and brain metastases were treated with weekly cisplatin (40–60 mg $m^{-2}$) during WBRT (50 Gy over 5 weeks). Three patients had a CR and two a PR to the treatment, and four were alive more than 6 months later. Also, Lange et al (1987) reported that 27 patients with NSCLC and brain metastases were treated with ifsofamide (daily for 5 days at 2 g $m^{-2}$) and BCNU (at 30 mg $m^{-2}$ on days 1,3 and 5) and radiotherapy was given simultaneously. Of the 27, seven patients had a CR and 12 a PR. The mean time of survival was 9.5 months for patients with NSCLC. In an uncontrolled study of patients with brain metastases from solid tumours, concurrent radiotherapy and chemotherapy appeared to increase the median survival, although no information was provided regarding the extent of extracranial metastatic disease or other prognostic factors.

In our study of concurrent WBRT and chemotherapy with cisplatin, vindesine and mitomycin for NSCLC with brain metastasis, the response rate of the brain lesion to treatment was 75.8% (including five patients with CRs), and the response rate of the primary lesion to treatment was 18%. Also, the treatment improved at least one grade of performance status in 30% and at least one grade of neurological functions in approximately 55% of the patients. Median survival was 8 months and the 1-year survival rate was 40%. Median survival duration and 1-year survival rate, in our study, were better than the median survival durations (3–6 months) and 1-year survival rates (approximately 20%) in the previously mentioned WBRT-alone trials (Chu and Hilaris, 1961; Order et al, 1968; West and Maor, 1980), although our study was uncontrolled and had a bias for patient selection.

The major toxicity in this pilot study was leucopenia. Late toxicity of concurrent WBRT and chemotherapy are becoming more important with the longer survival of patients with metastatic disease (DeAngelis et al, 1989). It may be because of the short duration of survival in our series that we have experienced no neurotoxicity. Brain metastasis was the only clinical site of symptoms in 15% of the patients in our study. Thus, in spite of concurrent radiotherapy and chemotherapy, up to two-thirds of the patients died of recurrence of their other lesions before recurrence of brain lesions.

We have shown the ability to safely administer concurrent WBRT and MVP chemotherapy for patients with NSCLC and brain metastasis. There is an excellent imaging response rate to brain tumours, associated with improvement of neurological function and notable survival rates at a median survival duration and at 1 year after treatment. To evaluate the true benefit and the best use (accounting for quality of life and toxicity) of this treatment modality, we are conducting a prospective multicentre randomized study comparing WBRT with or without concurrent chemotherapy for patients with NSCLC and brain metastasis.

© Cancer Research Campaign 1997

### ACKNOWLEDGEMENTS

This study was supported in part by a grant-in-aid for Cancer Research from the Ministry of Health and Welfare in Japan (62S-1, 2S, 1, 5S, 1, 8S-1). We appreciate the assistance of Ms Yuki Hirochi in typing the manuscript and data collection and Mr Toshiyuki Ijima in statistical analysis.

### REFERENCES

Allen JC, Kim JH and Packer RJ (1987) Neoadjuvant chemotherapy for newly diagnosed germ cell tumors of the central nervous system. *J Neurosurg* **67:** 65–70

Boogerd W, Dalesio O, Bais EM and Van Der Sande JJ (1992) Response of brain metastases from breast cancer to systemic chemotherapy. *Cancer* **69:** 972–980

Borgelt B, Gelber R, Kramer S, Brady LW, Chang CH, Davis LW, Perez CA and Henderrickson FR (1980) The palliation of brain metastases: final results of the first two studies by the radiation therapy oncology group. *Int J Radiat Oncol Biol Phys* **6:** 1–9

Chu FCH and Hilaris BB (1961) Value of radiation therapy in the management of intracranial metastases. *Cancer* **14:** 577–581

Cox DR (1971) Regression model and life-tables. *J R Stat Soc B* **34:** 187–202

Deangelis LM, Delattre JY and Posner JB (1989) Radiation-induced dementia in patients cured of brain metastases. *Neurology* **39:** 789–796

Furuse K, Kubota K, Kawahara M, Kodama N, Ogawara M, Akira M, Nakajima S, Takada M, Kushunoki Y, Negoro S, Matsui K, Masuda N, Takifuji N, Kudoh S, Nishioka M and Fukuoka M (1995) Phase II study of concurrent radiotherapy and chemotherapy for unresectable stage III non-small cell lung cancer. *J Clin Oncol* **13:** 869–875

Greig NH (1989) Brain tumors and the blood–tumor barrier. In *Implications of the Blood–Brain Barrier and Its Maniplation,* Vol. 2, Neuwelt EA (ed.), pp. 77–106. Plenum: New York.

Hidalgo V, Carlos D, Hidalgo of and Calvo FA (1987) Simultaneous radiotherapy and cis-platinum for the treatment of brain metastases. *Am J Clin Oncol* **10:** 205–209

Horton J, Baxter DH, and Olson KB (1971) The management of metastases to the brain by irradiation and corticosteroids. *Am J Roentgerol* **3:** 334–335

Kantarjian H, Farha PA, Spitzer G, Murphy WK and Valdivieso M (1984) Systemic combination chemotherapy qw primary treatment of brain metastasis from lung cancer. *South Med J* **77:** 426–430

Kasamatsu Y, Sawada M, Setoguchi J, Onodera H, Nakai M, Takemura S, Kondo M, Satomura Y, Hara H and Hayashi H (1990) The effect of combination chemotherapy with mitomycin C (MMC), vindesine (VDS), and cisplatin (CCDP) for brain metastasis from non-small cell lung cancer (Japanese). *Haigan* **30:** 159–165

Kornblith PP, Walker MD and Gassidy JR (1985) Treatment of metastatic cancer to brain. In *Cancer Principle and Practice of Oncology*, 2nd edn, Devita VT, Hellman S and Rosenberg SA (ed.), pp. 2099–2104. Lippincot: Philadelphia.

Kristjansen PG and Hansen HH (1988) Brain metastases from small cell lung cancer treated with combination chemotherapy. *Eur J Cancer Clin Oncol* **24:** 545–549

Lange of, Schlechtingen J, Haase KD and Scheef W (1987) Simultaneous radiotherapy and chemotherapy in the treatment of brain metastases of malignant solid tumours. *Int Clin Pharm Res* **7:** 427–432

Lee JS, Murphy WK, Glisson BS, Dhingra HM, Holoye PY and Hong WK (1989) Primary chemotherapy of brain metastasis in small-cell lung cancer. *J Clin Oncol* **7:** 916–922

Mantel N (1966) Evaluation of survival data and two new rank order statistics arising in its cinsideration. *Cancer Chemother Rep* **50:** 163–170

Newlands ES (1985) Chemotherapy for brain metastases. *Prog Exp Tumor Res* **29:** 167–176

Order SE, Hellman S, Von Essen CF and Kligerman MM (1968) Improvement in quality of survival following whole-brain irradiation to grain metastasis. *Radiology* **91:** 149–153

Patchell RA, Tibbs PA, Walsh JW, Dempsey RJ, Maruyama Y, Kryscio RJ, Markesbery WR, Macdonald JS and Young B (1990) A randomized trial of surgery in the treatment of single metastases to the brain. *N Engl J Med* **322:** 494–500

Postmus PE, Smit EF, Haaxma-Reiche H, Van Zandwijk N, Ardizzoni A, Quoix E, Kirkpatrick A, Sahmoud T and Giaccone G (1995) Tenoposide for brain metastases of small cell lung cancer: a phase II study. *J Clin Oncol* **13:** 660–665

Sen DK, Sivanesaratnan V, Chuah CY, CH'NG SL, Simgh J and Paramsothy M (1987) Cerebral metastases from choriocarcinoma, results of chemotherapy. *Acta Obstet Gynecol Scand* **66:** 425–428

Stewart DJ, Benvenuto JA, Leavens M, Hall SW, Benjamin RS, Plunkett W, Mccredie KB, Burgess MA and Loo TL (1979) Penetration of 3-deazuridine into human brain, intracerebral tumour and cerebral fluid. *Cancer Res* **39:** 4119–4122

Stewart DJ, LU K, Benjamin RS, Leavens ME, Luna M, Yap HY and Loo TL (1983) Concentration of vindesine in human intracerebral tumour and other tissues. *J Neuro-Oncol* **1:** 139–144

Stewart DJ, Richard MT, Hugenholtz H, Dennery J, Nundy D, Prior J, Montpetit V and Hopkins HS (1984) Penetration of teniposide (VM-26) into human intracerebral tumours. *J Neuro-Oncol* **2:** 133–139

Sundaresan N, Galicich JH and Beattie E (1983) Surgical treatment of brain metastasis from lung cancer. *J Neurosurg* **58:** 666–671

Twelves CJ, Souhami RL, Harper PG, ASH CM, Spiro SG, Earl HM, Tobias JS, Quinn H and Geddes DM (1990) The response of cerebral metastases in small cell lung cancer to systemic chemotherapy. *Br J Cancer* **61:** 147–150

Vecht CJ, Haaxma-Reiche H, Noordijk EM, Padberg GW, Voormolen JH, Hoekstra FH, Tans JT, Lambooij N, Metsaars JA and Wattendorff AR (1993) Treatment of single brain metastasis: radiotherapy alone or combined with neurosurgery. *Ann Neurol* **33:** 583–590

Walker MD, Alexander E JR, Hunt WE, Maccarty CS, Mahaley MS, Mealey J JR, Norrell HA, Owens G, Ransohoff J, Wilson CB, Gehan EA and Strike TA (1978) Evaluation of BCNU and/or radiotherapy in the treatment of anaplastic gliomas. *J Neurosurg* **49:** 333–343

Weed JG and Hammond GB (1980) Cerebral metastatic choriocarcinoma: intensive therapy and prognosis. *Obstet Gynecol* **55:** 89–94

West J and Maor M (1980) Intracranial metastases: behavioral patterns related to primary site and results of treatment by whole brain irradiation. *Int J Radiat Oncol Bil Phys* **6:** 11–15

Workman P (1986) The pharmacology of brain tumor chemotherapy. In *Tumours of the Brain,* Bleehen NM (ed.) pp. 183–200 Springer-Verlag: Berlin.

World Health Organization (1979) *WHO Handbook for Reporting Results of Cancer Treatment.* WHO Offset Publication No. 48: Geneva.

Yamamoto M and Furuse F (1993) Treatment of brain metastasis from lung cancer (in Japanese). *Kokyu* **12:** 1022–1027

 © Cancer Research Campaign 1997